# Massachusetts

**Published:** 1895-09

**Authors:** F. H. Osgood


### LEGISLATION.

### MASSACHUSETTS.

*Summary of the Law of Massachusetts in Relation to the Suppression of Contagious Diseases Among Domestic Animals.*

THE laws covering this matter were passed in 1894–95. Under the laws the entire supervision and control is placed in the hands of the board of five Cattle Commissioners, appointed by the Governor, each for a term of three years.

The Commission as a board have control of the entire State, and are given power to make orders and regulations covering the importation and quarantine of cattle, the transportation and treatment of cattle within the State, the matter of quarantine of suspected cases of disease, and the disinfection and cleaning of premises, and are given generally the power to take all steps necessary or expedient to suppress or prevent the introduction of contagious diseases among domestic animals, including glanders, farcy, contagious pleuro-pneumonia, tuberculosis, Texas fever, foot-and-mouth disease, rinderpest, hog cholera, and rabies, and such other diseases as the Commission may deem to be contagious.

Besides this general power vested in the Commission, each member thereof is individually given the power to enter all premises for the purpose of inspection, to condemn and destroy diseased or unwholesome meat and carcasses which they may find; to quarantine and examine all animals suspected of being affected with or having been exposed to contagious disease, and, if they find them diseased, to cause such animals to be destroyed and safely disposed of, and in such case no compensation is paid to the owner of the animal destroyed, unless it is destroyed as having been affected with tuberculosis. When such animals are killed, the full value at the time of condemnation, not exceeding the sum of $60, is to be paid by the State, provided the animal has been owned within the State six months continuously prior to its being killed, and also provided that the owner has not wilfully contributed to the spread of the disease. This is a change over the law of 1894, in that under that law the State paid one-half the value at the time of killing, without taking into consideration the fact that the animal was affected with the disease for which it was condemned.

The value of the animal is to be determined either by agreement with the Commissioners or by appraisal of two arbitrators, one to be selected by the Commission and one by the owner. These appraisers are required to be sworn. If the owner is not satisfied with the appraisal, or with the action of the Commission in destroying his animal, he is given the power to appeal to the courts to determine the existence of the disease and the value.

Besides the Cattle Commissioners, appointed by the Governor, there are local boards of health within each city or town, selected or appointed by the voters or city and town authorities. The boards are given the general control of the matter of contagious diseases among the animals in their city or town, and it is made their duty to see that all outbreaks of disease are suppressed, and that suspected cases are quarantined promptly and reported to the Commission. They are bound by law to obey all lawful orders and regulations issued by the Cattle Commission or any of its members. They are given power to make regulations within the limits of their city or town, to prevent the spread of contagious diseases among domestic animals, and to quarantine animals. Such quarantine must continue until removed by them or by the Cattle Commission or some of its members.

It is made the duty of every person within the State, who suspects or has reason to believe the existence of contagious disease among animals, immediately to report it to the Board of Health of their city or town. The Board of Health are to cause the animal to be examined immediately by an inspector or competent veterinarian, and, if the animal be diseased, to report it to the Cattle Commission.

There is a third class of persons appointed throughout the State whose business it is especially to watch outbreaks of disease. These are local inspectors, each city or town being required to appoint one or more such annually. The compensation of these inspectors is paid by the city or town, except that in cities or towns of less than $2,500,000 valuation, the State pays one-half of the compensation of such inspector, this being a new provision passed at the last session of the Legislature. These inspectors are required to be sworn, are made the agents of the Board of Health, and are bound under penalty to obey all orders and regulations of the Commission. It is made their duty to make a regular inspection of all neat cattle, sheep, and swine within the limits of their city or town. Prior to this year such inspection only related to neat cattle. They are also directed to inspect all other animals where they suspect that disease may exist, and also to inspect the stables and premises where domestic animals are kept. Upon their inspecting such animals, if they suspect any of them to be diseased, it is made their duty immediately to quarantine such animals by serving written notice of such fact upon the owner or person in possession, and also to send a copy of such quarantine order immediately to the Board of Cattle Commissioners, and give notice to the Board of Health of the city or town. These inspectors have no power to remove quarantine imposed by them. In case of the inspection of neat cattle, sheep, or swine, if they find no disease to be present, it is their duty to give the owner a certificate of such fact.

Prior to this year the expense of all quarantine was required to be borne by the owner; but under the recent law, where specific animals are quarantined, and the owner is forbidden to sell the product, the State bears the actual expenses of the quarantine after the tenth day from the imposing of such quarantine.

The proprietors of all slaughter-house establishments engaged in the business of slaughtering neat cattle, sheep, or swine are obliged to take out a license, which license permits them to slaughter upon particular days. It is made the duty of the local inspector to be present on these days, and to inspect, at the time of slaughter, all neat cattle, sheep, and swine, and, if he finds them to be affected with a contagious disease, to seize and destroy them; for which the owner receives no compensation. Prior to this year no such inspection was required in the case of sheep and swine.

Owners not regularly engaged in the business are allowed to slaughter any of their neat cattle on their own premises without a license; but in such case they must have an inspector present to inspect the animal which is to be dealt with, the same as in case of animals slaughtered at the slaughter-house. If the animal is less than six months old, or has received a certificate of health from an inspector within six months, such animal may be slaughtered by the owner on his own premises without such certificate.

Under the law existing prior to the act of this year the matter of the inspection of animals was left entirely within the control of the Commission. They were to determine the method to be used in making these inspections. Under this law the Commission determined that the only reliable method of detecting tuberculosis was by the injection of tuberculin. This inspection was made entirely by the board of agents specially appointed by them for that purpose, and was not put into the hands of the local inspectors. Under this regulation all animals quarantined as suspected of tuberculosis were tested with tuberculin ; and, in addition to this, the board began a systematic examination of all animals in the State, beginning by quarantining such animals by counties, testing the neat stock within the limits of each county with tuberculin, freeing those found to be exempt from the disease, and destroying those found affected with it. Under the law passed at the recent session of the Legislature, it was provided that "until the first day of June, 1896, the use of tuberculin as a diagnostic agent for the detection of the disease known as tuberculosis in domestic cattle shall be restricted to cattle brought into the Commonwealth from any point without its limits, and to all cattle held in quarantine at Brighton, Watertown, and Somerville ; provided, however, that tuberculin may be used as such diagnostic agent on any animal or animals in any other portion of the State, upon the consent in writing of the owner or person in possession thereof, and upon any animals condemned as tuberculous upon physical examination by a competent veterinarian." Under this restriction the Commission have been obliged to abandon the systematic examination of cattle. They still require all animals coming into the State to be so tested, unless they enter the State accompanied by a proper certificate that they have successfully passed such tuberculin test. All animals quarantined as tuberculous, on physical examination, are also subjected to the tuberculin test, and tests are made upon written application of the owner.

In brief, the only changes effected by the law of this last year are to extend the regular examination of sheep and swine, to provide for the payment of quarantine expenses in certain cases after ten days, to provide for the payment of the full actual value of animals destroyed as tuberculous, in place of paying one-half the sound value, and to retrict the use of tuberculin.

For the purpose of carrying out this law for the entire year of 1895, the Legislature appropriated the sum of $150,000.

F. H. Osgood, M.R.C.V.S.

